# Hierarchical phononic crystals for filtering multiple target frequencies of ultrasound

**DOI:** 10.1038/s41598-020-64234-7

**Published:** 2020-05-15

**Authors:** Ki Yong Lee, Wonju Jeon

**Affiliations:** Department of Mechanical Engineering, Korea Advanced Institute of Science and Technology, 291 Daehak-ro, Yuseong-gu, Daejeon 34141 Republic of Korea

**Keywords:** Mechanical engineering, Acoustics

## Abstract

Hierarchically structured phononic crystals are proposed for filtering multiple frequency bands. The advantages of using structural hierarchy come from its multiscale periodicity and the increased number of controllable parameters, which contribute to open multiple bandgaps in broadband frequency ranges and adjust the positions of those bandgaps. By deriving a transfer-matrix-based theoretical formula, hierarchical phononic crystals are designed that filter the frequency bands for randomly selected frequencies in the ultrasonic range of 20 kHz to 10 MHz. Their wave-filtering capability is demonstrated by using numerical simulations with consideration of material loss. By comparing the transmittance spectra of the hierarchical phononic crystals with those of conventional ones, the structural hierarchy of the former is shown to be advantageous in filtering multiple frequency bands.

## Introduction

A hierarchical structure has the architectural feature of successively embedded structural elements with different length scales. Such structures occur naturally in numerous biological materials such as bone^1,2^, wood^2,3^, nacre^4^ and gecko foot pads^5^, and they are used in engineered structures such as the Garabit Viaduct^6^ and the Eiffel Tower^7^. In spite of the structural complexity, hierarchical structures are used widely in various engineering problems because of their mechanical properties. Generally, materials in nature are rarely both tough and stiff^8^, but those two mutually exclusive mechanical properties can be realized simultaneously in hierarchically structured materials. In static problems, the structural role of multiscale hierarchy in providing beneficial mechanical properties is now well known^8–12^.

Recently, several studies have explored the advantages of using hierarchical structures in dynamic problems. For instance, in thermodynamics, it was reported that using structural hierarchy in a honeycomb structure improved the heat resistance and thermal anisotropy, and those properties could be controlled by manipulating the geometrical parameters of the hierarchical structure^13^. Another application of hierarchical structure that has been investigated is for opening broad acoustic and elastic bandgaps. In 2013, Zhang and To^14^ showed that phononic crystals (PCs) with a hierarchical structure have broader acoustic stopbands than do those without hierarchy; they highlighted that using structural hierarchy opens bandgaps in low-frequency ranges. Thereafter, broader elastic bandgaps were obtained by applying self-similar hierarchies to honeycomb PCs^15^, nacre-like composites^16^, hub–spoke metamaterials^17^ and cross-like porous metamaterials^17^. Geometrical modification for obtaining wave energy attenuation using acoustoelastic metamaterials was also explored for multiscale structures such as fractals^18^, spider-web structures^19^ and multiscale resonators^20^. A common feature of the aforementioned studies is that they realized broadband wave filtering based on multiscale periodicity of hierarchy. However, hierarchical structures are not just for manipulating bandgaps; they can also be used to realize double-negative effective properties regarding the mass density and Young’s modulus of one-dimensional (1D) infinite structures^21^.

Previously, Zhang and To^14^ investigated the wave-filtering capability of 1D hierarchical structures thoroughly for different hierarchy levels. They reported that (i) the overall bandwidth covered by the bandgaps of PCs with hierarchy is much broader than that of the conventional PCs and (ii) using a higher level of hierarchy opens broad bandgaps in lower-frequency ranges.

In the present study, we propose 1D hierarchical PCs (HPCs) for filtering an arbitrary set of multiple target frequency bands. Whereas Zhang and To^14^ performed forward analysis by investigating the wave-filtering capability of the hierarchical structures, we consider the inverse design of the hierarchical structures with an in-depth understanding of their multiple bandgaps via quasi-static homogenization theory. Unlike the forward analysis, the inverse design obeys design constraints such as the maximum thickness of the structure, the unit cell size, and the number of unit cells. In other words, the ultimate goal of the present study is to find the optimal internal hierarchical structures for given geometrical constraints.

Because increasing the hierarchy level increases the number of geometrical parameters in the HPCs, evaluating their bandgaps by using finite-element simulations would be time consuming. Instead, we use the transfer matrix method^22^ to derive the exact power transmission coefficient HPCs, and we use this fast and accurate method to perform parametric studies. We perform finite-element simulations only to validate our theoretical results and predict the wave-filtering capability of the designed structures when considering material loss in a viscoelastic material.

As well as designing hierarchical structures, we investigate the advantage of HPCs over conventional PCs in filtering multiple target frequency bands. By using a quantity defined as the filtering efficiency, which is a measure of whether one can achieve the desired wave-filtering capabilities by using HPCs or conventional PCs, we show that structural hierarchy is a key concept for handling wave problems and is not limited to solving static problems or thermodynamic problems.

## Results

### Geometry of HPCs

A PC^23–25^ is a man-made structure comprising a periodic arrangement of inclusions or voids in a matrix. This structure uses the physical properties of interference or Bragg scattering to create phononic bandgaps at which waves cannot pass through the structure. Because of their wave-filtering capability, PCs have various applications such as wave guiding, filtering, harvesting and confinement.

**Figure 1:**
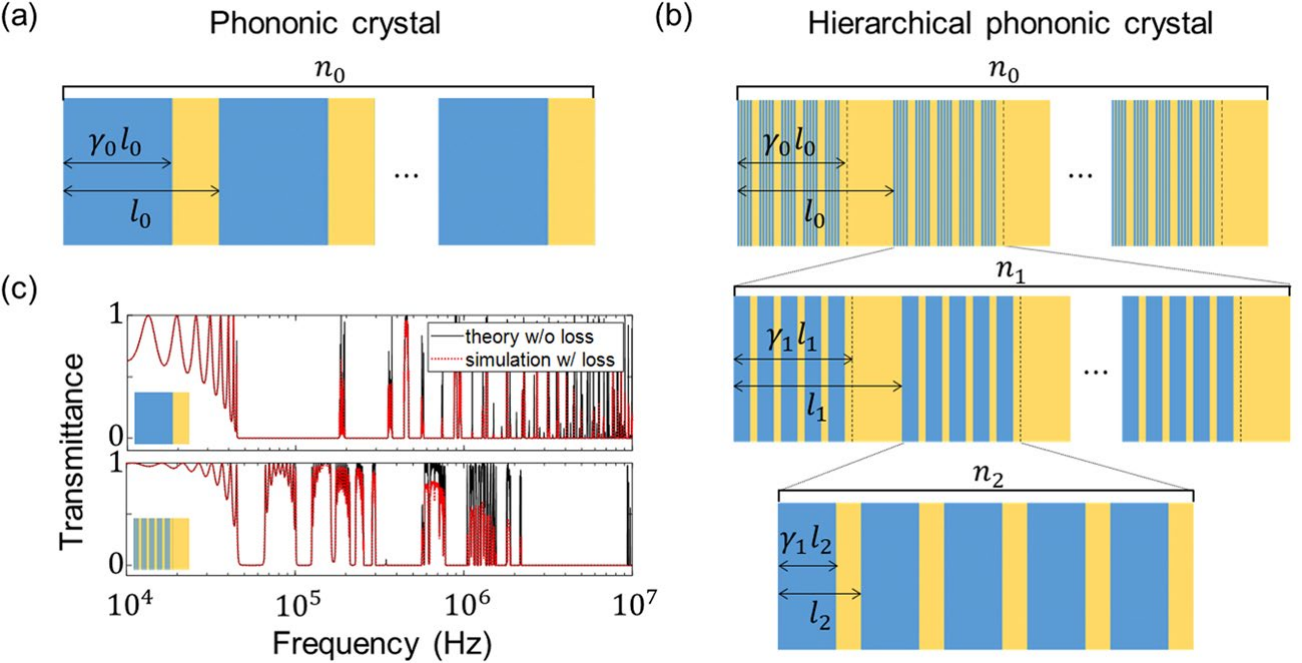
Geometry and transmittance spectra of one-dimensional two-phase phononic crystal (PC) and hierarchical PC (HPC). **(a)** PC with *n*_0_ = 10, *l*_0_ = 1 cm and *γ*_0_ = 0.7, and **(b)** HPC with hierarchy level *N* = 2, *l*_0_ = 1 cm, *n*_0_ = 10, *n*_1_ = *n*_2_ = 5 and *γ*_0_ = *γ*_1_ = *γ*_2_ = 0.7. Aluminium (blue) and PDMS (yellow) are used as the hard and soft materials, respectively. **(c)** Transmittance spectra of PC (upper) and HPC (lower). Black solid lines denote the theoretical results without loss and red dotted lines denote the numerical results with loss. The bandgaps in the frequency range below 10 MHz remain unchanged, which means that the material loss has almost no effect on the wave-filtering capabilities of the HPCs (or the conventional PCs) for the materials and geometrical parameters used in this study (i.e. viscoelastic PDMS and total thickness of less than 10 cm).

Figure 1a shows a 1D two-phase PC comprising hard (blue) and soft (yellow) materials whose filling fractions are *γ*_0_ and 1−*γ*_0_, respectively. Throughout this paper, aluminium (Al)^26^ and polydimethylsiloxane (PDMS)^27^ are used as the hard and soft materials, respectively, and their mass densities (*ρ*), longitudinal moduli (*κ*) and sound speeds (*c*) are given as follows: ${\rho }_{Al}=2,700\,{\rm{k}}{\rm{g}}/{{\rm{m}}}^{3},$ ${\rho }_{PDMS}=969\,{\rm{k}}{\rm{g}}/{{\rm{m}}}^{3}$, ${\kappa }_{PDMS}=1.21\,{\rm{G}}{\rm{P}}{\rm{a}}$, ${c}_{Al}=6,320\,{\rm{m}}/{\rm{s}}$ and ${c}_{PDMS}=1,119\,{\rm{m}}/{\rm{s}}$. Acoustic attenuation is considered with a frequency-dependent loss factor $\alpha =1.854\times {10}^{-7}\times {f}^{1.35}\,{\rm{d}}{\rm{B}}/{\rm{m}}$ for the viscoelastic medium PDMS^28^. The loss factor of Al is neglected because it is much smaller than that of PDMS.

As shown in Fig. 1b, an HPC is formed by successively embedding smaller scale of periodic structures into the locations of the hard material in a conventional PC. Here, *N* is the total number of hierarchy levels, *γ*_*i*_ is the filling fraction of the hard material, *l*_*i*_ is the size of the unit cell and *n*_*i*_ is the number of unit cells, with the subscript *i* indicating the geometrical parameters of the *i*-th level in the HPC. Figure 1b shows an example of an HPC with *N* = 2, ${l}_{0}=1\,{\rm{cm}}$, ${n}_{0}=10$, ${n}_{1}={n}_{2}=5$ and ${\gamma }_{0}={\gamma }_{1}={\gamma }_{2}=0.7$. From how the geometrical parameters are defined, the HPC becomes a conventional PC when *N* = 0, and the relation ${l}_{i}={\gamma }_{i-1}{l}_{i-1}/{n}_{i}$ holds for $i=1,2,\ldots N$.

In this study, we use only two materials, the geometrical parameters of which act as free variables (or controllable parameters) to satisfy our design goals. Of course, there are many ways to create the controllable parameters depending on how the hierarchical structure is constructed. First, if we use one hard material and one soft material, then the number of unit cells and the filling fraction for each hierarchy level are the controllable parameters. Second, if we use more than two constituent materials, then the additional degrees of freedom regarding the material selection for each layer can be considered. Third, in addition to the material selection and the manipulation of geometrical parameters, the desired wave-filtering capabilities can be achieved by changing how the materials are arranged in the hierarchical structure. Although the hierarchical structure could be designed with far more degrees of freedom, we restrict the number of materials to two because the main motivation of this study is to investigate whether one can achieve improved wave-filtering capabilities by changing only the geometrical framework from the conventional periodic structure to a hierarchical one, not by introducing any other materials or changing their arrangement.

### Multiple bandgaps of HPCs

We investigate the bandgap characteristics of HPCs by calculating their power transmission coefficients with the aid of a theoretical formula based on the transfer matrix method. In the section entitled ‘Methods’, we explain in detail the procedure for deriving the theoretical formula. Figure 1c shows the transmittance spectra of HPCs with the different hierarchy levels of *N* = 0 and *N* = 2. The black solid lines indicate the theoretical results without loss, and the red dotted lines indicate the numerical results with loss. As shown in Fig. 1c, the hierarchical structure opens a number of wide stopbands in the higher-frequency ranges because of the structural advantage of the multiscale periodicity, but the overall bandwidth of the stopbands in the lower-frequency ranges becomes narrow. In the figure, a couple of separate bandgaps in the high-frequency regime near 10 MHz are merged into a single stopband because of the acoustic attenuation. However, most bandgaps below 10 MHz remain unchanged, which means that considering the material loss has almost no effect on the bandgaps under the conditions of this study, namely using (i) the viscoelastic material PDMS, (ii) a total thickness of less than 10 cm and (iii) frequencies of less than 10 MHz.

To obtain a theoretical understanding of such HPC bandgap characteristic, we apply quasi-static homogenization theory. Homogenization is a method for evaluating the effective material properties of a heterogeneous medium. Especially in wave problems, when the wavelength of an incident wave is much longer than the size of a unit cell in a periodic structure, the effective mass density is obtained from the volume-weighted arithmetic mean of the mass densities, and the effective longitudinal modulus is obtained from the volume-weighted harmonic mean of the longitudinal moduli^29^. This homogenization technique that is suited to the long-wavelength limit is called quasi-static homogenization.

**Figure 2:**
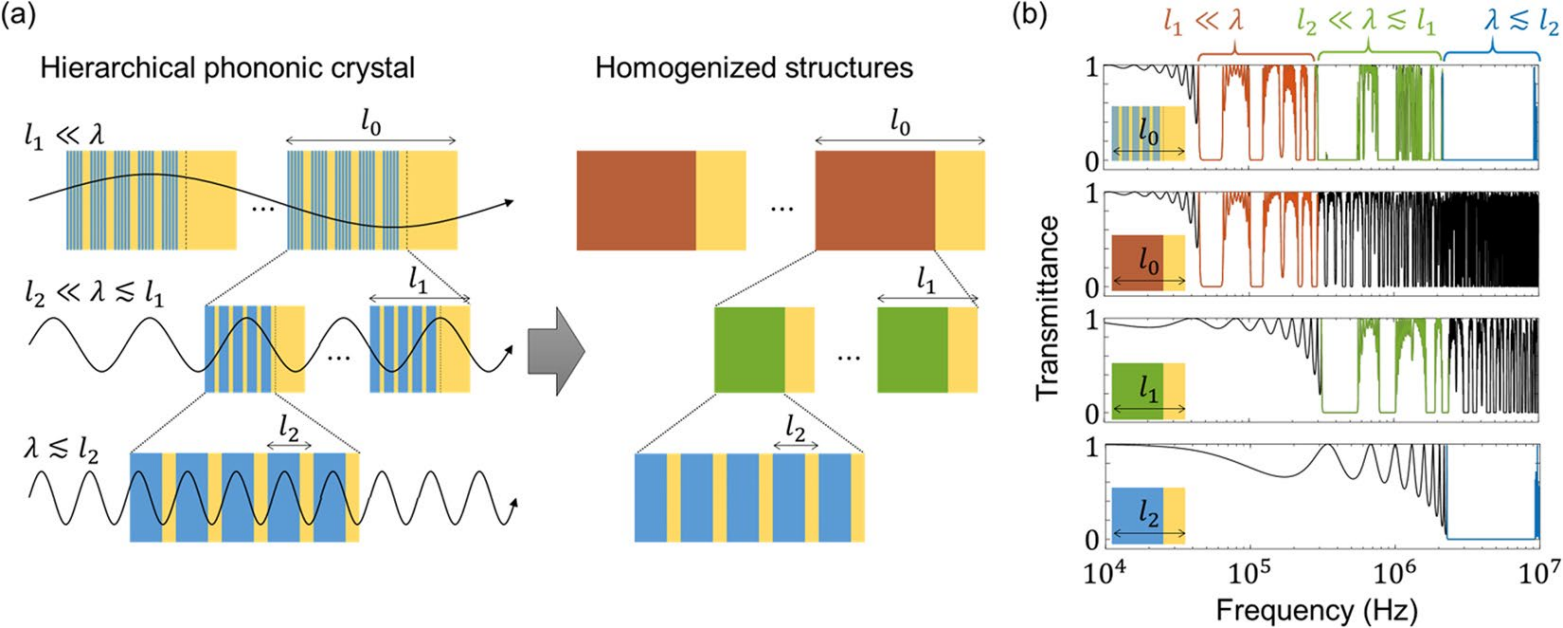
Relationship between bandgaps of HPC and homogenized structures. **(a)** Homogenized structures of HPC for different wavelengths. Depending on the wavelength, the HPC can be regarded as a different type of homogenized structure according to quasi-static homogenization theory. For example, if the wavelength is much larger than *l*_2_ but smaller than or comparable with *l*_1_, then the structure can be regarded as an *l*_1_*-*periodic homogenized structure comprising PDMS and a hard material with an effective density of ${\rho }_{eff}={\gamma }_{2}{\rho }_{Al}+(1-{\gamma }_{2}){\rho }_{PDMS}$ and an effective longitudinal wave modulus of ${\kappa }_{eff}={\{{\gamma }_{2}{\kappa }_{Al}^{-1}+(1-{\gamma }_{2}){\kappa }_{PDMS}^{-1}\}}^{-1}$. **(b)** Transmittance spectra of HPC and its homogenized structures. For each wavelength range, the stopbands of the homogenized structure accord with those of the HPC, which means that the multiple bandgaps of the hierarchical structure are the unions of bandgaps created by individual homogenized structures with periodicities corresponding to the different hierarchies.

Figure 2a shows *l*_0_*-, l*_1_*-* and *l*_2_*-*periodic homogenized structures for different wavelength scales. The HPCs can be regarded as different types of homogenized structure depending on the wavelength of the incident wave. For instance, when the wavelength is much larger than *l*_2_ and comparable with or smaller than *l*_1_, the HPC can be regarded as the *l*_1_*-*periodic homogenized structure comprising PDMS and an effective hard material with ${\rho }_{eff}={\gamma }_{2}{\rho }_{Al}+(1-{\gamma }_{2}){\rho }_{PDMS}$ and ${\kappa }_{eff}={\{{\gamma }_{2}{\kappa }_{Al}^{-1}+(1-{\gamma }_{2}){\kappa }_{PDMS}^{-1}\}}^{-1}$. Then, we can expect the bandgaps of the HPC in the wavelength range of ${l}_{2}\ll \lambda \lesssim {l}_{1}$ to be almost the same as those of the *l*_1_*-*periodic homogenized structure.

Figure 2b shows the transmittance spectra of the HPC and the homogenized structures. For each frequency range indicated by the different colours of red, green and blue, the transmittance spectra of the HPC and the homogenized structures are nearly same, which means that the bandgaps of the HPC are the union of those in the homogenized structures, and therefore multiple bandgaps occur in the hierarchical structure.

**Figure 3:**
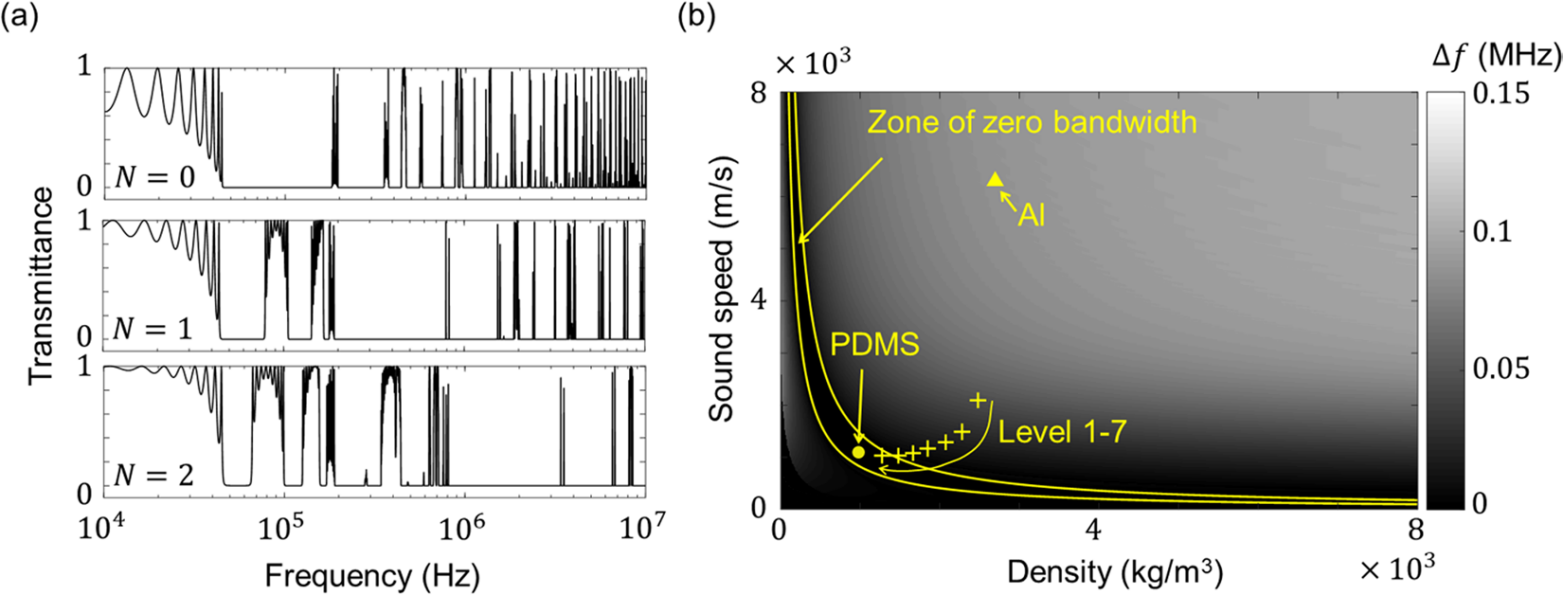
Effects of hierarchy level on multiple bandgaps. **(a)** Transmittance spectra of HPCs for different hierarchy levels of *N* = 0, *N* = 1 and *N* = 2. For the respective structures, the geometrical parameters are taken as $\{{l}_{0}=1\,{\rm{c}}{\rm{m}},\,{n}_{0}=10,\,{\gamma }_{0}=0.7\}$, $\{{l}_{0}=1\,{\rm{c}}{\rm{m}},\,{n}_{0}=10,\,{n}_{1}=3,\,{\gamma }_{0}={\gamma }_{1}=0.7\}$ and $\{{l}_{0}=1\,{\rm{c}}{\rm{m}},\,{n}_{0}=10,\,{n}_{1}={n}_{2}=3,\,{\gamma }_{0}={\gamma }_{1}={\gamma }_{2}=0.7\}$. The frequency ranges covered by the stopbands are much broader for higher hierarchy levels, but the first stopband range is narrowed. **(b)** Bandwidth of first stopband (Δ*f*) of periodic structures for different hard materials with fixed soft material of PDMS. The crosses indicate the homogenized properties of the hard materials in the *l*_0_*-*periodic scale, and the yellow triangle and circle denote the material properties of Al and PDMS, respectively. As the hierarchy level increases, the yellow crosses approach the zone of zero bandwidth and Δ*f* becomes null for hierarchy levels higher than six, which means that an unnecessarily high level of hierarchy has no merit for acoustic wave filtering.

Furthermore, we investigate why the relative bandwidth of the first stopband in the HPC is smaller than that of the conventional PC. In general, the relative bandwidth of the first stopband in PCs increases with increasing impedance contrast between the constituent materials. Thus, for the *l*_0_*-*periodic homogenized structure, the relative bandwidth of the first stopband decreases because the impedance contrast decreases as the hierarchy level increases. Because the first stopband of the *l*_0_*-*periodic structure is almost the same as that of the HPC (as shown in Fig. 3b), the relative bandwidth of the first stopband in the HPC also decreases as the hierarchy level increases. From the investigation via quasi-static homogenization, using a hierarchical structure is advantageous in broadening the phononic bandgaps but diminishes the wave-filtering capability in the low-frequency range.

### Effects of geometrical parameters on phononic bandgaps

In this section, we investigate how the geometrical parameters of an HPC affect its multiple bandgaps. Of the four geometrical parameters *N*, *γ*_*j*_*, l*_*j*_ and *n*_*j*_, we consider mainly the effects of *N* and *γ*_*j*_ because those of the other two parameters are already well established and can be understood from Eqs. (9)–(11). In Eqs. (9)–(11), the term *l*_*j*_ is always multiplied by the wavenumber *k*_*j*_, which means that the frequency ranges of the bandgaps are inversely proportional to *l*_*j*_. Meanwhile, the number *n*_*j*_ of unit cells is proportional to the frequency ranges of the bandgaps in the case of *j* ≠ 0 because the following relation holds for *j* ≥ 0: ${l}_{j+1}={\gamma }_{j}{l}_{j}/{n}_{j+1}$. In the case of *j* = 0, we require *n*_0_ ≥ 3 for the stopbands to exist.

Figure 3a shows the transmittance spectra of the HPCs with the different hierarchy levels of *N* = 0, *N* = 1 and *N* = 2. In the respective cases, the geometrical parameters are given by $\{{l}_{0}=1\,{\rm{c}}{\rm{m}},\,{n}_{0}=10,\,{\gamma }_{0}=0.7\}$, $\{{l}_{0}=1\,{\rm{c}}{\rm{m}},{n}_{0}=10,{n}_{1}=3,{\gamma }_{0}={\gamma }_{1}=0.7\}$ and $\{{l}_{0}=1\,{\rm{c}}{\rm{m}},{n}_{0}=10,{n}_{1}={n}_{2}=3,{\gamma }_{0}={\gamma }_{1}={\gamma }_{2}=0.7\}$. As shown in the figure, the HPC with the highest hierarchy level has multiple broad bandgaps in the frequency ranges of 40 kHz to 10 MHz. Results such as these mean that an HPC with a higher hierarchy level is more suitable for filtering multiple frequencies in a broad frequency range.

However, increasing the hierarchy level decreases the bandwidth of the first stopband, thereby weakening the wave-filtering capability in the low-frequency range. To find the hierarchy level at which the first stopband begins to disappear, we investigate the first stopbands of HPCs with different hierarchy levels. The first stopband of an HPC can be obtained by calculating that of the *l*_0_*-*periodic homogenized structure, and Fig. 3b shows the bandwidth of the first stopband with respect to the sound speed in the hard material and its mass density. The yellow triangle and circle indicate the material properties of Al and PDMS, respectively, and the yellow crosses indicate the effective properties of the hard material in the *l*_0_*-*periodic homogenized structure for different hierarchy levels from one to seven.

When the hard-material properties are in the zero-bandwidth zone bounded by the two yellow lines indicating $z=0.8\,{\rm{M}}{\rm{R}}{\rm{a}}{\rm{y}}{\rm{l}}$ and $z=1.4\,{\rm{M}}{\rm{R}}{\rm{a}}{\rm{y}}{\rm{l}}$, the first stopband becomes null. As the hierarchy level increases, the yellow crosses approach the zero-bandwidth zone, and an HPC with a hierarchy level of at least six has no stopband. An interesting point is that many hierarchically structured biomaterials that possess an extraordinary resistance to waves have a hierarchy level of six^1,2,9,10^. Those observations could be interpreted as meaning that hard biomaterials have adopted six as their optimal hierarchy level to protect themselves from the catastrophic failure by dynamic attack^30–32^.

**Figure 4:**
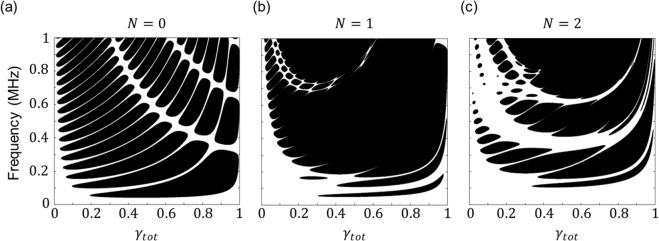
Effects of total filling fraction (*γ*_*tot*_) on phononic bandgaps. Bandgaps of HPCs for different total filling fractions of Al for **(a)** *N* = 0, **(b)** *N* = 1 and **(c)** *N* = 2. The black and white areas indicate stopbands and passbands, respectively. For each structure, the filling fractions of the embedded structures are identical, which means ${\gamma }_{0}={\gamma }_{1}={\gamma }_{tot}^{1/2}$ in the case of *N* = 1. The other geometrical parameters are taken as $\{{l}_{0}=1\,{\rm{c}}{\rm{m}},{n}_{0}=10\}$, $\{{l}_{0}=1\,{\rm{c}}{\rm{m}},{n}_{0}=10,{n}_{1}=3\}$, and $\{{l}_{0}=1\,{\rm{c}}{\rm{m}},{n}_{0}=10,{n}_{1}={n}_{2}=3\}$ for the respective structures.

In what follows, we investigate how the total filling fraction (*γ*_*tot*_) affects the bandgaps of HPCs. Figures 4a–c show the bandgap regions of the HPCs (*N* = 0,1,2) for different total filling fractions of Al. The filling fraction is set as ${\gamma }_{i}={\gamma }_{tot}^{1/(N+1)}$ in each hierarchical level; for instance, in the case of $N=2$, we have ${\gamma }_{0}={\gamma }_{1}={\gamma }_{2}={\gamma }_{tot}^{1/3}$. The other geometrical parameters are taken as $\{{l}_{0}=1\,{\rm{c}}{\rm{m}},{n}_{0}=10\}$, $\{{l}_{0}=1\,{\rm{c}}{\rm{m}},{n}_{0}=10,{n}_{0}=3\}$ and $\{{l}_{0}=1\,{\rm{c}}{\rm{m}},\,{n}_{0}=10,\,{n}_{1}={n}_{2}=3\}$ for the respective hierarchical structures. The black areas indicate the stopbands and the white areas indicate the passbands.

As shown in Fig. 4, the HPCs with smaller total filling fraction have either narrow bandgaps or no stopband because of the small impedance contrast between the hard and soft materials of the ${l}_{0}$-periodic structure. Increasing ${\gamma }_{tot}$ opens several bandgaps, and for ${\gamma }_{tot}$ >  0.5 the HPCs have a couple of narrow bandgaps in the low-frequency range but broad bandgaps in the high-frequency range. The positions of the multiple bandgaps in HPCs are highly dependent on the filling fraction.

### HPCs for filtering multiple frequencies

In this section, we compare the filtering efficiency of HPCs with that of conventional PCs. The ‘filtering efficiency’ is a measure of the possibility that one could design (or obtain) a proper structure that filters an arbitrary set of target frequency bands; it is defined as the ratio of the number of filtered frequency sets to the total number of test sets. In this study, we generate 2,000 sets of frequencies for 10 repetitions, thus we examine 20,000 test sets for each number of target frequencies. Here, a frequency is considered as ‘filtered’ when the power transmission coefficient is less than 10^−4^.

**Figure 5:**
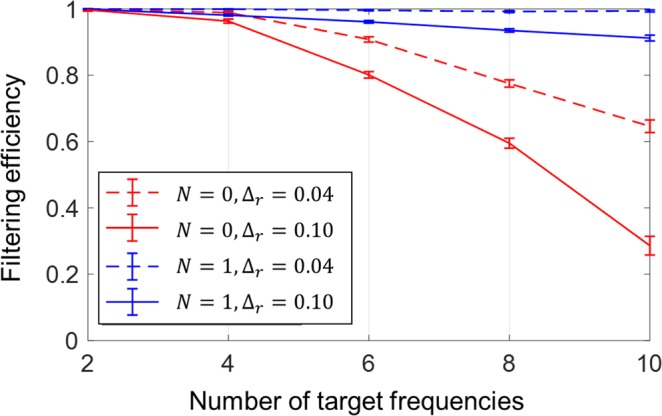
Filtering efficiencies of HPCs for hierarchy levels of 0 and 1. Red and blue lines indicate the filtering efficiencies of PCs (i.e., *N* = 0) and hierarchical structures (i.e., *N* = 1), respectively. Dashed and solid lines denote the results for relative bandwidths of 0.04 and 0.10, respectively. Each error bar represents ±2 standard deviations, and its centre is the mean filtering efficiency evaluated for 10 repetitions. Each repetition has 2,000 sets of randomly generated frequencies in the ultrasonic frequency range of 20 kHz to 10 MHz for each number of target frequencies.

The frequency sets used to evaluate the filtering efficiency are generated using the ‘rand’ function in the commercial software MATLAB R2018b, which is a function for generating uniformly distributed random numbers. For given numbers *R*_*n*_ and *R*_*s*_, we generate a set of *R*_*n*_ random numbers within the open interval (0, 1) for *R*_*s*_ repetitions, so that the random numbers in each test set are distributed almost uniformly. Then, for each test set, we use a linear mapping to transform those random numbers within (0, 1) to the range of (20 kHz, 10 MHz) to obtain the target frequencies. In this study, we use *R*_*n*_ = 2,000 and *R*_*s*_ = 10, and thus the standard deviations for *R*_*s*_ sets are indicated by error bars for each number of target frequencies, each hierarchy level and the relative bandwidth, as shown in Fig. 5.

Figure 5 shows the filtering efficiency of the HPCs with respect to the number of target frequencies for different hierarchy levels and relative bandwidths. The red lines indicate the filtering efficiency of PCs (i.e. *N* = 0), and the blue lines indicate the filtering efficiency of HPCs (i.e. *N* = 1). The dashed and solid lines denote the results for the relative bandwidths of 0.04 and 0.10, respectively. Each error bar represents plus or minus two standard deviations, and its centre is the mean value of the filtering efficiency evaluated for 10 repetitions. As shown in Fig. 5, the filtering efficiency of the conventional PCs with *N* = 0 decreases faster with increasing number of target frequencies compared to the case of *N* = 1. When the number of target frequencies is 10 and the relative bandwidth is 0.1, the filtering efficiency is only 0.286 in the case of *N* = 0 but is over 0.9 in the case of *N* = 1. These results show that HPCs have much better capability for frequency filtering than do conventional PCs, especially for a large number of target frequencies. For example, we design one-level HPCs to filter 10 target frequencies that conventional PCs cannot filter, namely 0.095, 0.27, 0.42, 0.66, 1.21, 1.96, 2.48, 4.38, 5.58 and 8.61 MHz.

**Figure 6:**
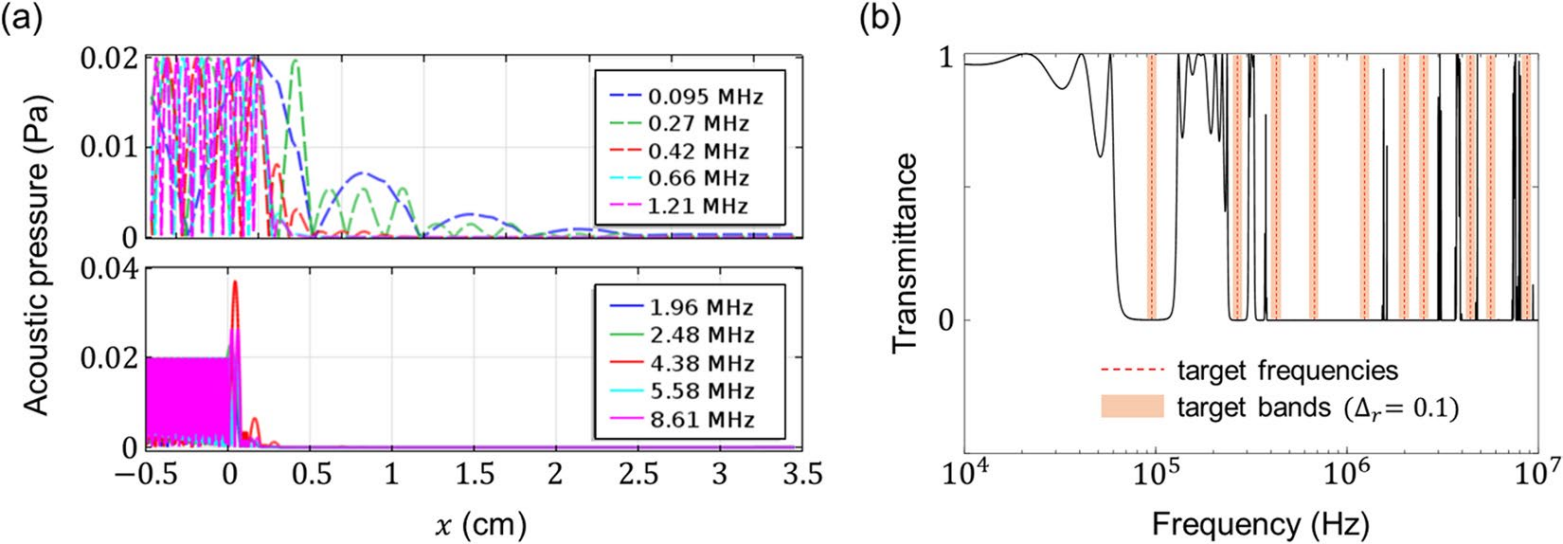
Wave-filtering capability of designed HPC for given target frequencies and relative bandwidth. **(a)** Acoustic pressure in designed structure $(0\,{\rm{cm}}\le x\le 2.7\,{\rm{cm}})$ and outer water medium ($x\le 0\,{\rm{cm}}$ or $x\ge 2.7\,{\rm{c}}{\rm{m}}$). The sound source located at $x=-0.5\,{\rm{c}}{\rm{m}}$ emits longitudinal waves with the target frequencies and an amplitude of 0.01 Pa in the positive *x* direction. The acoustic pressure at $x\ge 2.7\,{\rm{cm}}$ is nearly zero in all cases. **(b)** Transmittance spectrum of the designed HPC. The red dashed lines indicate the target frequencies and the orange areas indicate the target frequency bands with a relative bandwidth of Δ_*r*_ = 0.1. All the target frequencies with bands fall into the stopbands, thereby showing that the wave-filtering capability of the designed structure is realized well.

By following the design procedure given in the section entitled ‘Methods’, an HPC with the geometrical parameters of $\{{l}_{0}=0.65\,{\rm{c}}{\rm{m}},{\gamma }_{0}=0.55,{\gamma }_{1}=0.7,{n}_{0}=4,{n}_{1}=3\}$ is designed to filter the 10 frequencies with a relative bandwidth of ${\varDelta }_{r}=0.1$. To demonstrate the wave-filtering capability of the designed hierarchical structure, we investigate the acoustic pressure distribution and transmittance spectrum when a longitudinal wave with an amplitude of 0.01 Pa is incident on the designed structure, as shown in Figs. 6a,b. The results in Fig. 6a show that the acoustic pressure decreases sharply near the left interface ($x=0\,{\rm{c}}{\rm{m}}$) between the outer fluid and the designed structure, and the acoustic pressure is almost zero at the right interface ($x=2.7\,{\rm{c}}{\rm{m}}$), indicating that the longitudinal waves with the 10 targeted frequencies are filtered perfectly in the structure. In addition, as shown in Fig. 6b, the transmittance is almost zero in the targeted bands (${\varDelta }_{r}=0.1$) whose centre frequencies are the 10 targeted frequencies, implying that the designed HPCs can filter not only the target frequencies but also the targeted bands.

In the design procedure, the number of unit cells and the filling fractions at each level were used as the control parameters. Additional degrees of freedom could be obtained by inserting a third material at different hierarchy levels or changing the structural arrangements, but we used only two materials because the ultimate goal of the design procedure was to find the optimal internal structure for each hierarchy level with the simplest combinations of two types of material.

## Discussion

In this study, PCs with structural hierarchy were designed to have multiple bandgaps in a broad frequency range by calculating their exact power transmission coefficient via the transfer matrix method for a 1D multi-layered structure. The overall bandwidth covered by multiple bandgaps using the HPCs was an order of magnitude broader than that of a conventional PC with single periodicity. The multiple bandgaps in the HPCs were characterized as the union of bandgaps created by multiscale periodicities based on quasi-static homogenization. Among the geometrical parameters affecting the phononic bandgaps, an HPC with hierarchy level *N* of greater than six has no bandgap because the contrast in acoustic impedance between the soft material and the homogenized hard material decreases after several homogenization steps for each scale, and our interpretation is that unnecessarily high level of hierarchy gave no further improvement in wave-filtering capability.

We compared the filtering efficiencies of hierarchical structures and conventional periodic structures and found that the former are more capable of filtering several wide frequency bands. The relative bandwidth was considered in the design procedure to retain the wave-filtering capability of the hierarchical structures even if the target frequencies are shifted within a small range because of uncertainty. In addition, the wave-filtering capability of the designed structure was demonstrated by using numerical simulation with material losses.

To conclude, we mention briefly our ongoing work on HPCs. Because all the results in the present study were obtained for 1D HPCs, we cannot guarantee the wave-filtering capability of hierarchical structures for higher-dimensional issues such as oblique incidence, shear vertical waves, or curved geometries. To bridge the gap between the concept of structural hierarchy and its actual applications, we are extending 1D HPCs to higher-dimensional ones comprising two or more materials by considering their geometrical arrangements. As discussed in earlier sections, introducing different combinations of materials at different hierarchy levels or changing their structural arrangement are possible ways to create the additional degrees of freedom needed to design advanced-level hierarchical structures. We are using the acoustic–solid interaction module in COMSOL for the numerical computations to consider elastic deformations and the propagation of shear vertical waves. Our ongoing work also includes fabricating HPCs made of Al and PDMS and investigating their ultrasound-blocking capability in water. Because the hierarchical structures in the present study are made from continuous materials whereas those in previous studies were mostly structured with discrete materials, the technical challenges of fabricating our hierarchical structures must be addressed. We fabricate by using overlay-aligned roll-transfer printing^33^, which is suitable for fabricating multi-layered hierarchical structures.

## Methods

### Power transmission coefficients of HPCs

The power transmission coefficient of an HPC is derived by using the transfer matrix method for 1D multi-layered structures. When a harmonic plane wave propagates in a 1D stratified medium, the displacement and stress fields are governed by

1 $$\left(\frac{{d}^{2}}{d{x}^{2}}+k{(x)}^{2}\right)u(x)=0,$$

2 $$-\rho (x){\omega }^{2}u(x)=\frac{d}{dx}\sigma (x),$$

where *k* is the wave number, *u* is the displacement field, *ρ* is the mass density, *ω* is the angular frequency and *σ* is the stress field. A general solution for Eq. (1) expresses the displacement field *u*_*j*_(*x*) in the *j*-th layer as the summation of traveling waves in the positive *x* direction, namely ${u}_{j}^{+}(x)={A}_{j}^{+}{e}^{i{k}_{j}x}$, and the negative *x* direction, namely ${u}_{j}^{-}(x)={A}_{j}^{-}{e}^{-i{k}_{j}x}$. The stress field ${\sigma }_{j}(x)$ in the *j*-th layer is evaluated by substituting the displacement field *u*_*j*_(*x*) into Eq. (2). The displacement and stress fields in the *j*-th layer are expressed in matrix form as

3 $$[\begin{array}{c}{u}_{j}(x)\\ {\sigma }_{j}(x)\end{array}]={{\bf{P}}}_{j}{{\boldsymbol{Q}}}_{j}(x)[\begin{array}{c}{A}_{j}^{+}\\ {A}_{j}^{-}\end{array}],$$

where $${{\bf{P}}}_{j}=[\begin{array}{cc}1 & 1\\ i\omega {z}_{j} & -i\omega {z}_{j}\end{array}]$$ and $${{\boldsymbol{Q}}}_{j}(x)=[\begin{array}{cc}{e}^{i{k}_{j}x} & 0\\ 0 & {e}^{-i{k}_{j}x}\end{array}]$$.

In Eq. (3), *z*_*j*_ is the characteristic impedance of the material in the *j*-th layer. Let ${x}_{j}^{L}$ and ${x}_{j}^{R}$ be the left and right boundary positions, respectively, of the *j*-th layer. Considering the equality ${x}_{j}^{R}={x}_{j}^{L}+{s}_{j}$ that holds for the thickness *s*_*j*_ of the *j*-th layer, the relation between the displacements and stresses at the left and right boundaries is derived as

4 $$[\begin{array}{c}u({x}_{j}^{R})\\ \sigma ({x}_{j}^{R})\end{array}]={{\boldsymbol{M}}}_{j}[\begin{array}{c}u({x}_{j}^{L})\\ \sigma ({x}_{j}^{L})\end{array}],$$

where ${{\boldsymbol{M}}}_{j}={{\boldsymbol{P}}}_{j}{{\boldsymbol{Q}}}_{j}({s}_{j}){{\boldsymbol{P}}}_{j}^{-1}$ is the transfer matrix for the *j*-th layer and is expressed by

5 $${{\boldsymbol{M}}}_{j}=[\begin{array}{cc}\cos ({k}_{j}{s}_{j}) & \sin ({k}_{j}{s}_{j})/(\omega {z}_{j})\\ -\omega {z}_{j}\,\sin ({k}_{j}{s}_{j}) & \cos ({k}_{j}{s}_{j})\end{array}].$$

Applying the continuity conditions of displacement and stress to each boundary, the displacement and stress at the left boundary of the first layer are the same as those at the right boundary of final *J*-th layer with a cumulative transfer matrix ${\boldsymbol{M}}={{\boldsymbol{M}}}_{J}{{\boldsymbol{M}}}_{J-1}\cdots {{\boldsymbol{M}}}_{1}$, namely

6 $$[\begin{array}{c}u({x}_{J}^{R})\\ \sigma ({x}_{J}^{R})\end{array}]={\boldsymbol{M}}[\begin{array}{c}u({x}_{1}^{L})\\ \sigma ({x}_{1}^{L})\end{array}].$$

Combining Eqs. (6) and (3) for *j* = 0 and *j* = *J* + 1, the relation between the coefficients of traveling waves in the left and right outer media is derived as

7 $$[\begin{array}{c}{A}_{J+1}^{+}{e}^{i{k}_{n+1}{s}_{t}}\\ 0\end{array}]={\boldsymbol{\Omega }}[\begin{array}{c}{A}_{0}^{+}\\ {A}_{0}^{-}\end{array}],$$

where ${s}_{t}=\mathop{\sum }\limits_{j=1}^{J}{s}_{j}$ and ${\boldsymbol{\Omega }}={{\boldsymbol{P}}}_{J+1}^{-1}{\boldsymbol{M}}{{\boldsymbol{P}}}_{0}$. The power reflection coefficient of a multi-layered structure is defined as the square of the ratio between the amplitudes of the propagating and reflected waves in the left outer medium, and therefore the power transmission coefficient is expressed by

8 $$T=1-{|\frac{{A}_{0}^{-}}{{A}_{0}^{+}}|}^{2}=1-{|\frac{{\Omega }_{21}}{{\Omega }_{22}}|}^{2}.$$

An advantage of the transfer matrix method is that the reflectance and transmittance of a multi-layered medium can be calculated simply by multiplying matrices in terms of the geometrical parameters and the constituent materials. We use this method to derive the power transmission coefficient of an HPC. Denoting ***M***^(*N*)^ as the cumulative transfer matrix of an HPC with a hierarchy level of *N*, we have that ***M***^(0)^, ***M***^(1)^ and ***M***^(2)^ are expressed as

9 $${{\boldsymbol{M}}}^{(0)}={({{\boldsymbol{M}}}_{S}^{(0)}{{\boldsymbol{M}}}_{H}^{(0)})}^{{n}_{0}},$$

10 $${{\boldsymbol{M}}}^{(1)}={({{\boldsymbol{M}}}_{S}^{(0)}{({{\boldsymbol{M}}}_{S}^{(1)}{{\boldsymbol{M}}}_{H}^{(1)})}^{{n}_{1}})}^{{n}_{0}},$$

11 $${{\boldsymbol{M}}}^{(2)}={({{\boldsymbol{M}}}_{S}^{(0)}{({{\boldsymbol{M}}}_{S}^{(1)}{({{\boldsymbol{M}}}_{S}^{(2)}{{\boldsymbol{M}}}_{H}^{(2)})}^{{n}_{2}})}^{{n}_{1}})}^{{n}_{0}},$$

where $${{\boldsymbol{M}}}_{S}^{(j)}=[\begin{array}{cc}\cos ({k}_{S}(1-{\gamma }_{j}){l}_{j}) & \sin ({k}_{S}(1-{\gamma }_{j}){l}_{j})/(\omega {z}_{S})\\ -\omega {z}_{S}\,\sin ({k}_{S}(1-{\gamma }_{j}){l}_{j}) & \cos ({k}_{S}(1-{\gamma }_{j}){l}_{j})\end{array}]$$ and $${{\boldsymbol{M}}}_{H}^{(j)}=[\begin{array}{cc}\cos ({k}_{H}{\gamma }_{j}{l}_{j}) & \sin ({k}_{H}{\gamma }_{j}{l}_{j})/(\omega {z}_{H})\,\\ -\omega {z}_{H}\,\sin ({k}_{H}{\gamma }_{j}{l}_{j}) & \cos ({k}_{H}{\gamma }_{j}{l}_{j})\end{array}]$$.

Here, the subscripts *H* and *S* stand for the hard and soft materials, respectively. Comparing Eqs. (9) and (10) shows that ***M***^(1)^ is the same as ***M***^(0)^ if ${{\boldsymbol{M}}}_{H}^{(0)}$ in Eq. (9) is substituted into ${({{\boldsymbol{M}}}_{S}^{(1)}{{\boldsymbol{M}}}_{H}^{(1)})}^{{n}_{1}}$. Similarly, comparing Eqs. (10) and (11) shows that ***M***^(2)^ is the same as ***M***^**(**1)^ when ${{\boldsymbol{M}}}_{H}^{(1)}$ in Eq. (10) is substituted into ${({{\boldsymbol{M}}}_{S}^{(2)}{{\boldsymbol{M}}}_{H}^{(2)})}^{{n}_{2}}$. Repeating those operations gives the cumulative transfer matrix for an HPC of level *N* as

12 $${{\boldsymbol{M}}}^{(N)}={({{\boldsymbol{M}}}_{S}^{(0)}{({{\boldsymbol{M}}}_{S}^{(1)}(\cdots {({{\boldsymbol{M}}}_{S}^{(N-1)}{({{\boldsymbol{M}}}_{S}^{(N)}{{\boldsymbol{M}}}_{H}^{(N)})}^{{n}_{N}})}^{{n}_{N-1}}\cdots ))}^{{n}_{1}})}^{{n}_{0}}.$$

By putting Eq. (12) into Eqs. (7) and (8) gives the exact power transmission coefficient of an HPC with a hierarchy level of *N*.

Here, we demonstrate the applicability of the theoretical formulas in Eqs. (7), (8), (12) by comparing its calculated power transmission coefficients with those from numerical simulations for different hierarchy levels. As shown in Fig. 1c, the theoretical results agree well with the numerical results. Also, the computational time was reduced by a factor of around 2,000 compared to the numerical simulations. This made it easy to manipulate the bandgaps and find the optimal structure by controlling several geometrical parameters in the hierarchical structures, and we obtained the statistical data in Fig. 5 through repeated calculations for random sets of target frequencies.

### Procedure for designing HPCs

In this section, we summarize the four-step procedure for designing HPCs to filter multiple frequency bands.

Step 1. We choose the maximum thickness of the entire structure (3 cm in the present study), the constituent materials (Al and PDMS in the present study) and the outer medium (water in the present study).

Step 2. We choose the hierarchy levels (0 and 1 in the present study), the numbers of target frequencies (2, 4, 6, 8 and 10 in the present study) and the relative bandwidths for the target frequencies (0.04 and 0.1 in the present study). For the given numbers of target frequencies and the relative bandwidths, we choose multiple target frequencies randomly from the ultrasonic frequency range of 20 kHz to 10 MHz. The detailed procedure for generating frequency sets randomly is explained in the second paragraph of the section entitled ‘HPCs for filtering multiple target frequencies’.

Step 3. We obtain the transmittance spectra of the hierarchical structures by changing the free parameters of *l*_0_ (thickness of the unit cell in the 0-th hierarchy level), *γ*_*i*_ (filling fraction of Al in the *i-*th hierarchy level) and *n*_*i*_ (number of unit cells in the *i-*th hierarchy level). The total thickness of the hierarchical structure (i.e. *n*_0_*l*_0_) is set to be less than or equal to the maximum thickness of 3 cm. Those free parameters are shown in detail in Fig. 1b. For a parametric sweep, we use the sufficiently small resolutions of 1 mm for *l*_0_ and 0.001 for *γ*_*i*_, and we use the maximum value of 10 for *n*_*i*_ to avoid designs that cannot be fabricated, such as a layer that is thinner than the manufacturing precision.

Step 4. From the results obtained from the parametric sweep in step 3, we select sets of geometrical parameters such that the hierarchical structure with those parameters filters the union of all the frequency bands centred at each target frequency with its relative bandwidth. Here, the design criterion regarding the frequency filtering is that the power transmission coefficients be less than 10^−4^.

By following the above design procedure, we obtain HPCs that filter multiple target frequency bands for given materials and geometrical constraints.

### Numerical simulations

We used the finite-element method (COMSOL 5.2a software) to calculate the acoustic pressure distribution in each designed HPC and its outer medium. The governing equation was the 1D Helmholtz equation for harmonic analysis, and we considered the frequency-dependent loss of PDMS. The computational domain was [−0.5 cm, 3.5 cm], the designed structure was located at [0 cm, 2.7 cm] and the rest of the domain as water. The element size was determined so that there were 12 nodes for the shortest wavelength. The sound source located at *x* = −0.5 cm emitted time-harmonic plane waves in the positive x direction. Plane-wave radiation conditions were given at both ends of the computational domain to prevent unphysical reflections from the boundaries of the domain.
